# Resilience of environmental policy amidst the rise of conservative populism

**DOI:** 10.1007/s13412-021-00721-1

**Published:** 2021-09-22

**Authors:** Nariman Mostafavi, João Fiocchi, Manuel García Dellacasa, Simi Hoque

**Affiliations:** 1 Department of Civil, Architectural and Environmental Engineering, Drexel University, 3141 Chestnut Street, Curtis 251, Philadelphia, PA 19104 USA; 2 Department of Sociology, University of Pennsylvania, 3718 Locust Walk, McNeil 313, Philadelphia, PA 19104 USA; 3 Department of Economics, University of Massachusetts, 412 North Pleasant Street, Crotty Hall, Amherst, MA 01002 USA

**Keywords:** Environmental protection, Conservative populism, Environmental policy, Resilience, Neoliberalism

## Abstract

Sustainability has for long been promoted as a medium for social and economic development, one that focuses on constant availability of natural assets and ecological amenities. By questioning the possibility of reaching a balanced and sustainable state of functioning for social-ecological systems, resilience improves the static framework of sustainability by acknowledging non-linear behavior of complex systems, inevitability of change, and consistent presence of uncertainty. At the core of sustainable development, environmental policy is embedded in the socio-spatial structures that constantly re-organize and breed uncertainty, such as political, economic, and climate uncertainty. These uncertainties create episodes of instability that shock the entire system including the structures of environmental protection. In this article, focusing on the aftermath of 2016 US presidential election and 2018 general election in Brazil, both broadly recognized as political shocks, we highlight the vulnerabilities of environmental protection structures to the rise of conservative populist movements. We attribute these vulnerabilities, partially, to the superiority of market-based instruments, as well as apolitical understandings of resilience under neoliberalism that overlook political instabilities and socio-spatial outcomes of neoliberal restructuring projects. In our assessment, political unpreparedness of sustainability against the right-wing onslaught in the US and Brazil further underlines the need for resilience theory to incorporate sources of political instability in order to protect the environment.

## Introduction

Achieving sustainability, as a societal ideal and a development objective, has mostly centered conservation of natural resources and responsible management of ecological services to guarantee future availability, through reaching a stable equilibrium state between supply and demand (Moore et al. 2017). However, despite partial triumphs of sustainability as a comprehensive environmental governance model, many of its goals—curbing climate change, for instance—remain largely out of reach (Abson et al. 2017). It is now widely argued that aspiring to a stable equilibrium state for the operation of complex social ecological systems has been nonviable from the start.

Resilience discourse is a response to the more recent reckonings that planning is no longer feasible without embracing uncertainty and recognizing the possibility of system functioning in more than a single, stable equilibrium state. It accepts the inevitability of shocks and acknowledges the nonlinear behavior of complex systems and the possibility of regime change (Walker and Salt 2006; Swanstrom et al. 2009). Social sciences and public policy fields have adopted resilience frameworks from natural science disciplines to prepare social networks and communities, as well as evaluating their preparedness to face major uncertainties (Carpenter et al. 2005; Brand 2009). Uncertainties breed incidents of shock—commonly referred to as disasters—that sometimes surprise the system so distinctively, leading to policy crisis episodes. The resilience discourse promotes preparing for those uncertainties in order to avoid, mitigate, manage, and recover from the disaster strikes. However, policy crises that follow shocking incidents are strongly linked to how resilience is understood.

It is easy to miss the role that relations of power play in determining what constitutes crisis, threat, or disaster (Hudson 2009). Power relations are shaped by various interactions among groups and individuals with different and often conflicting vested interests and varying degrees of influence. Political and economic outcomes of these interactions are heavily influenced by the balance of power between competing interest groups (Grazhevska 2019). Change in the balance of power can bring about reorganization phases or instability episodes that impact, inter alia, environmental policy and sustainability agendas. Therefore, apolitical interpretations of resilience not only implicitly advocate for preserving existing structures and insulating the interests of privileged interest groups from social transformations, but also fail to capture systemic susceptibilities to political uncertainties.

The global victories of populist conservative movements^1^ post 2016, initially framed as political *shocks*, have had major consequences for local and global environmental protection structures since sustainability agenda and legislating for environmental protection are implicated and reshaped in intrinsically political socio-economical processes. The emerging conservative administrations and legislators have successfully rolled back many environmental laws and regulations at both national and international levels^2^ and therefore, have seriously questioned the durability and resilience of the environmental protection social and legal structures. Throughout this paper we suggest there is a correlation between the lack of preparedness of the sustainability promoting structures against political attacks from populist conservative movements and the steady trust in the effectiveness of market mechanisms in protecting the environment, as well as overlooking the threats lying in the political arena. In the following sections, we discuss the connections between apolitical understandings of resilience and the neoliberal philosophy, explore the vulnerabilities of environmental law to conservative populist movements by focusing on the USA and Brazil cases after 2016 and 2018 elections respectively, and argue that improving the resilience of environmental policy structures requires systemic understanding of power relations and political dynamics at different scales.

## Politics of environmental protection

Environmental protection is at the core of sustainability discourse that underlines equitable social and economic growth while preserving natural resources for future generations (Jasrotia 2007). Transitioning to sustainability requires visioning, leadership, and experimentation, encouraged and facilitated by transition management governance approaches (Smith and Stirling 2010). Both areas of resilience and sustainability have unique intellectual histories and problem framing backgrounds, rooted in, also galvanizing different politics with many contrasts and similarities. The politics of social and environmental transformation are pronounced through regimes of truth, rule, and accumulation (Watts and Peluso 2013). These overlapping regimes postulate power and articulate political dynamics in connected, yet different ways. Regimes of truth inspire perceptions on knowledge, society, threat, technology, history, and culture. Regimes of rule determine hegemonic reigns of knowledge and practice and the positionality of political actors, in other words, formers and controllers of governance. Finally, regimes of accumulation regulate relations of production and distribution (Scoones 2016).

The power dynamics and political developments impacting environmental policy cannot be fully fathomed outside the realms of neoliberal political economy. In the globalized world, regimes of truth, rule, and accumulation implicating and reshaping politics of environmental protection are increasingly dominated by neoliberalism (Jessop 2002; Gledhill 2018). Over the last half century, market mechanisms have taken over power centers in decision-making structures, including environmental policy (Peck et al. 2009). Governance entities have been challenged by increased neoliberalization and entrepreneurialization as economies get deregulated, public-sector missions and responsibilities are privatized and decentralized, and workfare-based social approaches replace welfare state obligations (Eraydin 2013; Purcell 2009; Leitner et al. 2007). Besides unprecedented wealth and income inequality, the combination of uncontrolled market-based production and unrestrained consumerism has led to over-consumption and grotesque pollution levels of natural ecosystems. Competition as the driver of the accumulation model, and competitiveness as the means of survival are constantly escalating interregional and international conflicts (Chatagnier and Kavaklı 2017). Furthermore, due to the asymmetrical neoliberal restructuring of spatial relations, local actors are left with increasingly less power and more responsibility to respond to a wide range of crises, as opposed to wealthy multinational players who have constantly gained more power and freedom (Peck and Tickell 2002; Neilson 2014).

The neoliberal psyche prescribes a shift towards self-regulatory structures by building legal values and normative limitations into decision-making processes and developing “corporate conscience” (Selznick 1994, p. 345) and, as a result, reducing the role of state in environmental policy- making. Although some would argue that “social license” demands (Gunningham et al. 2004) and symbolic commitments of self-regulating can leverage the normative aspirations underlying environmental law and achieve enhanced outcomes and compliance practices (Short and Toffel 2010), it is clear that the role of the state as regulatory enforcer cannot be entirely replaced by self-regulation. In the US, for instance, before the consolidation of neoliberalism, environmental regulating duties were assigned to the federal government while the states predominantly preserved permitting and enforcing powers. The Clean Air Act (CAA), the National Environmental Policy Act (NEPA), the Federal Water Pollution Control Act (Clean Water Act), the Safe Drinking Water Act, the Toxic Substances Control Act, the Resource Conservation and Recovery Act, and the Comprehensive Environmental Response, Compensation, and Liability Act, which are marker statutes that fostered the centralization of environmental rulemaking, all got passed through 1963–1980, the period in which the federal government was still powerful enough to confront the polluting corporations. That “federal authority over environmental policy also avoided the much-feared ‘race to the bottom,’ in which polluters would shop around for lenient states willing to sacrifice environmental quality for new jobs and economic growth (Harrington et al. 2004, p. 244).”

Since the 1980s, however, large numbers of think-tanks, corporations, and foundations have been advocating for market solutions for environmental protection, aspiring to insert neoliberal theory into the environmental discourse by investing in public outreach projects and cooperating with interest groups, bureaucrats, and lobbyists. The Institute of Economic Affairs in the UK and the Heritage Foundation, Political Economy Research Centre, Competitive Enterprise Institute (CEI) and the Science and Environmental Policy Project (SEPP) in the US, for decades, have been promoting free market environmentalism and economic liberalism (laissez-faire libertarianism), advertising that privatization, deregulation, and free market solutions are more than enough to address environmental problems (Beder 2001). The influence of neoliberal ideas popularized by these thinktanks has been rife. Payments for Ecosystem Services (PES), biodiversity offsets, and carbon and other pollutions trading schemes are some of the market-based policy instruments that have been promoted as more flexible and cost-effective alternatives to traditional *command-and-control* approaches (Pirard 2012). The establishment of tradable property rights for ecosystem services as an environmental policy solution stems from doctrines that consider environmental conservation pointless or at least impossible unless it is profitable. Even some environmental advocacy groups not particularly known for being *conservative*, such as the Natural Resources Defense Council (NRDC) and the Environmental Defense Fund (EDF) have strongly pushed for tradeable pollution rights for years.

Public regulation failures in reversing the rapid downturn of natural ecosystems and the assumptions that market forces somehow rise above politics play a key role in the popularity of market-based instruments (Sandel 2012). In addition, the influence of think-tanks and businesses in mainstreaming market approaches is seldom consciously acknowledged. In the opening ceremony of the World Commission on Environment and Development in 1985, Rakel Surlien—former Norwegian Minister of Environment—said that “we must be willing to examine our relations in international trade, investments, development assistance, industry, and agriculture in light of the consequences these may have for underdevelopment and environmental destruction in the Third World.” However, in 1987, in accordance with Thatcher and Reagan convictions, Bruntland sustainable development report demanded “more rapid economic growth …, freer market access for the products of developing countries, … and significantly larger capital flows, both concessional and commercial (WCED 1987, article 72).” The 1992 Rio Declaration emphasized that “trade policy measures for environmental purposes should not constitute a means of arbitrary or unjustifiable discrimination or a disguised restriction on international trade” and “national authorities should endeavour to promote the internalization of environmental costs and the use of economic instruments, taking into account the approach that the polluter should, in principle, bear the cost of pollution, with due regard to the public interest and without distorting international trade and investment (UNCED 1992).” The efforts to make the case for further liberalization of trade as *the* stimulator of sustainable development and economic growth and warnings to avoid any inconsistencies with WTO rules continued in the 2002 Johannesburg Declaration on Sustainable Development and the Earth Summit 2012. In the 1990s Europe favored direct regulation and taxation instruments, until intense industrial lobbying and the US withdrawal from the Kyoto Protocol saw them also adopt the trading system in 2001 (Spash 2010). Some strongly believe that in the US, corporations led the government towards pollution trading schemes in order to maximize their profits and minimize the chances of ever being required to control their pollutions (Lohmann 2006). But in fact, more unusual than the rule of market-based instruments in the US is the nearly perfect consensus on their superiority over regulatory standards. Such widespread approval cannot be the product of business lobbying toil only. A broad alliance of government agencies, academic institutions, and think-tanks have urged for the application of trade mechanisms in environmental policy for decades (Gómez-Baggethun and Muradian 2015), illustrating the aforementioned constitution of the regimes of truth shaping decision-making structures.

It is hard to deny that environmental international policymaking is undergoing a stage of instability as well. Multinational environmental policy efforts have failed to stop or even impede climate change or loss of biodiversity trends. After a few significant agreements created by successful international negotiations such as 1987 Montreal Protocol on ozone layer depletion, 1994 Vienna Convention of Nuclear Safety, 1997 Kyoto GHG reduction Protocol, and the 2000 Cartagena Protocol on biosafety, a phase of *treaty congestion* and *treaty fatigue* is noticeable until the 2016 Paris Agreement. This slow-down should not have been surprising. Emerging economies like China and Brazil have been imposing themselves as veto players while the US is retreating from leadership roles, hampering the process of reaching multilateral agreements. In addition, international environmental institutions are terribly under-budgeted compared to trade or health organizations and in many cases have been undermined by the liberal mandates of other international institutions.

Furthermore, the environmental degradation phenomena have a widening gap across the Global North–South division lines. The appropriation of the environmental resources upon which developing economies depend can hardly be separated from the expanding markets and technology networks. Foreign investments have been directed towards developing countries whenever they can purvey conveniences such as low-cost labor or natural resources. The development pattern that prioritizes specific ecologies over others has brought about shrinkage of commons areas; expansion of pasture, timber and crop lands; privatization of ubiquitous resources; vast spreads of hazardous environments; and compromised self-determination capacities of developing countries, and has sensibly been referred to as environmental colonialism (Byrne et al. 2002), disproportionately impacting marginalized resource-dependent populations. The technological breakthrough, however, has not necessarily carried the advancements to the less developed corners of the world. Instead of eradicating poverty or inequality, some even speculate that new technologies are implemented to further exploit the Global South (Omamo and von Grebmer 2005; Love 2003). There are arguments suggesting that while developed countries have enjoyed over a century’s worth of heavily fossil fuel intensive growth, the under-developed countries’ equitable access to these natural resources they vitally need is denied through environmental regulation (e.g., Soomin and Shirley 2009). From a postcolonial and southern perspective, the environmental agenda of the Global North promoted by international organizations could also be perceived to violate environmental sovereignty of less developed countries, dampen the voices of local citizenry through top-down policy structures, and entangle small economies in quagmires of international bureaucracies. The very same concept, ecological imperialism, has alternatively been explained as deliberate ecological transformation of the weaker economies in terms of introduction of new industries, land use, and management practices by developed countries with devastating impacts on indigenous communities and their natural environments (Crosby 2015) in order to maintain the flow of raw materials into the global production machine.

## Environmental policy and conservative movements

Conservative movements have historically advocated for market-based instruments, and against centralization of environmental policy making, under the pretext of defending free trade, property rights, and individual freedoms. They also have largely been doubtful of environmental international multilateralism. Unilateralism endorsed by Strauss advises political interest groups to conduct their own policy and stay away from international organizations. In his opinion, “for the foreseeable future, political society remains what it always has been: a partial or particular society whose most urgent and primary task is its self-preservation and whose highest task is its self-improvement (Strauss 1964),” and therefore, survival is only (or ought to be) achieved through independence and self-protection. Internationalism has been widely regarded as a threat to democracy and independence by conservative movements. Some American conservatives, for instance, go as far as saying that after FDR, American democracy was transformed into a “cooperative commonwealth,” and not “free, honorable, independent” anymore (Kesler 2009). Conservative British philosopher, Roger Scruton, defends “local initiatives against global schemes, civil association against political activism, and small-scale institutions of friendship against large-scale and purpose-driven campaigns (Scruton 2012).” He criticizes the liberal framing of free enterprise as an assault on natural resources and attributing the environmental damages to big businesses and the economies within which they thrive, arguing that upon hypothetical abolishment of the market economy, the same destruction would occur, this time with the hands of the states instead of corporations. In his opinion, in an economy under the rule of law, predations of businesses can be more easily restricted (by sovereign governments) than those of powerful states, although he acknowledges that “law must keep pace with the threats” to reduce the environmental cost of economic freedom. Scruton maintains that *oikophilia*—love of household—can be the only valid foundation for environmentalism that can actually produce results. Similarities between Scruton’s ideas and slogans of the contemporary conservative movements are abundant. They emphasize reinvigoration of national stewardship, sentimentalize the countryside life, and blame the state and regulations for the decline of the old prosperous rural life, not the big supermarkets, developers, or technology. The environmental policy arising from this stance is “think locally, act nationally.”

Deepak Lal, neoliberal economist, suggests that an internationally just distribution of resources is not achievable since no universally accepted set of moral values underpin this posture, and global public goods cannot be distributed worldwide analogous to welfare states in the West. He maintains that for dealing with professed global public goods issues (e.g., the side effects of transnational transport pollutions), the capacity of the existing international organization (e.g., UNEP and WHO) is more than enough, and there is no need for additional multi-layer global institutions and that “the demands for a new global financial architecture are misguided (Lal 2000).” In his own words, “… paradoxically far from being an expression of American imperialism, the current period of globalization is threatened by the reluctance of the US to maintain its PAX, while at the same time attempting to legislate its ‘habits of the heart’ through a form of ‘ethical imperialism’.” Neoconservative Straussians justify and advocate *benevolent hegemony* of the American imperialism since it improves the national security and strategically serves the nation’s interest. The benign imperialism offered by neoconservatives “ought to emphasize both personal and national responsibility, relish the opportunity for national engagement, embrace the possibility of national greatness, and restore a sense of the heroic (Kristol and Kagan 1996).” This agenda, having reached perfection in the *America First* rhetoric, has pushed the US in a direction that can strangely be conceptualized as *isolationist imperialism*. It is now openly stated that the eternal goal of policy is national interest and security and of course not compassion propagation or protecting the environment of *others*. This is not new. Socrates, in Plato’s Republic, does not have a problem with imperialist expansionist war, whenever such conquest is *necessary* to guarantee the flow of sustaining materials into the city. Among the more contemporary advocates is Thomas G. West, American professor of politics and vocal supporter of global populist right movements, who suggests that some human societies (Iraq in his stance) lack “minimal democratic virtues of personal self-restraint and feisty self-assertion in defense of liberty, along with a widespread belief in the moral and/or religious obligation of everyone to respect the equal rights of others to life, liberty (including the free exercise of religion), and property (West 2004).” The emerging shade of conservatism like its predecessors posits that military interventions are appropriate to settle conflicts, but is different in the way it regards post-war *nation-building* efforts unnecessary. This breaking with predecessors is framed as taking *liberal* out of *liberal hegemony* (Posen 2018).

The inability of international institutions in containing conflicts that are only natural to human society and US’ unwillingness to step up to the challenge will “continue to put strains on the globalized economy through their spillover effects in the form of refugees as well as the direct economic costs in the countries/regions concerned,” in Lal’s view. In addition, globalization could face even more resentment if understood as a process of ethical imperialism through which international institutions enforce values upon and control regional governments, what Huntington sees as a backlash against secular, classical, liberal Western views (Huntington 1993). Those views often imply that any attempt for taming the process of globalization or interruptions to the nearly perfect free flow of capital, whether caused by ideological doctrines, self-determination tendencies, or financial crises, would imperil the purported era of perpetual peace and prosperity. However, with regard to environmental policy, radical environmentalists share the skepticism of conservatives on the efficacy of international multilateralism, despite accordance with liberals on an international outlook, averring that the international diplomatic structures and market-based instruments have extremely limited capacity to tackle the global environmental crisis which itself is a symptom of the greater capitalism’s existential crisis (Falkner 2013). Overall, the dismissal of international treaty-based environmental policy is either due to its alleged unenforceable distractive nature or its non-systemic, non-transformative agenda, by conservatives and progressive environmentalists respectively.

Despite arguments asserting the ideology of environmentalism orthogonal to classic conservatism-liberalism cultures, conservatism has been more negative towards pro-environmental views and movements. The involvement of government in protecting the natural resources goes against a core aspect of conservatism: economic libertarianism. Still, regardless of the criticisms from the private sector, industrial capitalism has not been sabotaged by air and water pollution control regulations (McCright and Dunlap 2003). On the contrary, it has failed to address the wide range of crises via market-led technological transitions. Julian Simon, a conservative environmental economist, rejected any significant correlations between population growth and famine, suggesting that the worldwide steady drop in the number of hungry people from 1970 to 1995 would continue thanks to the unburdened capacity of human talent and technological ingenuity (Nicholson 2015). In spite of his free-market and technological optimism, worldwide hunger has been continuously on the rise ever since (Margulis 2013) in a backward move (which he did not live to see). Comparably, ecological modernization theory suggests that further industrialization and advancement of technology not only are by no means problematic, but are the most reliable options for escaping the environmental crisis (Spaargaren and Mol 1992). The theory depicts environmental progress as economically feasible and advocates for new coalitions for making it politically feasible as well (Fisher and Freudenburg 2001). However, the idea of a “sustainable capitalism” (O’Connor 1994) has been harshly criticized for being “not possible” and lacking a recognizable set of postulates (Buttel 2000; York et al. 2010).

By and large, in contrast to conservative movements, more liberal political movements have failed to define specific crystallized goals, only promising endless transformation and progression. This lack of final mission best symbolizes itself in open-ended ambiguous campaign slogans such as *hope* and *change* and can in return be challenged by all the vagueness in phrases such as *great again*. Today, amidst the global rise of conservative populism, the powerful and vigorous arrangement of enterprising bureaucracy and extensive metaregulation is sliding into, or at least majorly threatened by a novel version of regressive and fatal antiregulation drive of Reagan/Thatcher days. The ineffective crusade of the center-leaning liberal governments to establish sustainable regulatory structures and irreversibly restore welfare state services, anticompetitive organizations, and intervention capacities of government agencies is in some cases succeeded by a reactionary phase of dominance of the shallowest of the neoliberal political representatives who prioritize destruction of the ruling statecraft over creating an alternative governing model. The world has been struggling to understand the causes and implications of the substantial recent victories of conservative populist right that took many pundits by surprise. The emerging conservative administrations have been quite effective in rolling back some of the major environmental regulations in favor of business stakeholders and undermining international environmental regulatory structures. In the rest of this section we briefly discuss the consequences of these political developments in the US and Brazil, as two notable case studies.

Our methodology is mostly aligned with comparative historical analysis methods. Such methods (i) are concerned with causal analysis (Ragin 1987; Munck 1998; Mahoney 1999; Mahoney and Rueschemeyer 2003); (ii) study processes over time (Abbot 1990, 1992; Aminzade 1992; Pierson 2000a, 2000b; Rueschemeyer and Stephens 1997; Mahoney and Rueschemeyer 2003); and (iii) rely on contextualized and systematic comparisons (Ragin 1987, 2000; Locke and Thelen 1995; Mahoney and Rueschemeyer 2003). We examine the changes in environmental policy after the 2016 US presidential election (until 2020) and the 2018 general election in Brazil, both broadly perceived as political shocks at their time of happening. Therefore, we observe patterns by looking at both countries’ environmental legislations, environmental mandates (normative instructions, decrees, and ordinances), news from credible media outlets, and secondary literature. The objective is to contextualize and assess the impact of the rise of conservative populism on environmental protection systems and examine how resilient these systems have been in the face of conservative populism.

### The case of the USA

In the US, environmental policy-making has grown intense and strongly partisan with time, leading to increased congressional deadlock. The void of hard to pass environmental regulations is partially filled by litigation (Morriss et al. 2004). Moreover, as moving regulation through Congress has gotten more difficult, implementing executive authority has become profoundly relevant to impose environmental policy. The configuration of the US administrative state has developed as a perplexing maze of institutions and bureaucracies with different agendas and forms of influence that question the power of the federal Congress as the sole gateway to the environmental regulation process at the national level. Such unique arrangement of policy routes can be seen as the reaction of the classic neoliberal state obsessed with deregulation to its need to constant growth, stability, and accumulation of capital. This rare hodgepodge of contradictory motives has also slashed the efficiency and continuity of alternative policy paths in endless political disputes. For example, the attempt by the Obama administration to use executive authority in order to regulate carbon emissions came under a multitude of Congressional attacks through bills, amendments, and Continuing Resolutions such as Defending America’s Affordable Energy and Jobs Act, No More Excuses Energy Act, Free Industry Act, and EPA Stationary Source Regulations Suspension Act. Eventually, enforcement of the Clean Power Plan under CAA was halted by the Supreme Court on February 9, 2016. Congressional conservatives have continuously put forth bills that would eliminate civic litigation in environmental statutes such as NEPA and CAA. Ceaselessly, counter suits have been getting filed to revoke or at least delay the implementation of environmental policies. Vigorous onslaughts launched by the conservative legislature over the past decade have also aimed at overturning many progressive environmental regulations at subnational levels. For instance, the Climate Protection Act was eventually repealed in Florida, so were the Global Warming Response Act in New Jersey, and the Clean Energy Jobs Act in Wisconsin. Oregon, Washington, Montana, Utah, Arizona, and New Mexico have all dropped out of the Western Climate Initiative (WCI) (see MacNeil 2017).

The number of US environmental policies that never got challenged in courtrooms is very small. American litigation has harmed regulatory productivity for the most part and has caused relaxation and delay of many environmental rules. Proposed policies are disputed in regulatory proceedings and commonly challenged in federal district courts, circuit courts, and even the Supreme Court after enactment (Harrington et al. 2004). The story of NEPA itself is a perfect example that exhibits the importance of judicial interpretation for the enforceability of agency mandates. Signed into law in 1970 when concerns for the environment and optimism towards human environmental interventions were at coincident peaks, its extensive authority across US environmental regulatory regimes was so broad as it “assumed quasi-constitutional status as one of the foundational laws of the modern administrative state (Karkkainen 2004).” NEPA was enacted to prompt US government policy “use all practicable means and measures, including financial and technical assistance, in a manner calculated to foster and promote the general welfare, to create and maintain conditions under which man and nature can exist in productive harmony” and “fulfill the social, economic, and other requirements of present and future generations of Americans” and require all agencies’ actions to be “environmentally sound,” and “mandating that all laws, policies and regulations of the federal government conform to that policy.” However, the spirited objectives of NEPA were undermined by the interpretation of the courts, holding that NEPA Sect. 101 should not be understood beyond an inspirational assertion since it lacks the details required for enforcement. As a result, NEPA’s intentions were reduced to requiring agencies to take a set of procedural steps (including providing an Environmental Impact Statement) and seriously consider the impacts of their actions on the environment, without having to take any specific actions to counter those impacts (Benson and Garmestani 2011; Kalen 2008). Necessarily, as a legal matter, it has been the “procedure and not substance that matters in environmental impact assessment in the United States (Karkkainen 2005).”

After Donald J. Trump’s victory in 2016, presidential executive authority not only was wrecked as a tool for advancing environmental policy, but also became an effective instrument for undoing much of the progress previously made. The President Obama’s flood standards for federal infrastructure projects, the ban on offshore oil and gas drilling in the Atlantic and Arctic oceans, and the northern Bering Sea region protection rule all got revoked by sweeping presidential executive orders “promoting energy independence and economic growth.” Through transforming the Environmental Protection Agency (EPA) and the Interior Department procedures the new administration managed to weaken land and wildlife protections and loosen regulations designed to control toxic emissions. For instance, the EPA effectively suspended the Clean Water Rule and lowered the emissions standards for cars and light duty trucks that were considered by the administration to have been made with “politically charged expediency.” Furthermore, the Republican controlled Congress passed a bill allowing coal companies to dump mining debris into local streams, and the Keystone XL and Dakota Access pipelines got approved by the administration. These changes were not unexpected. As a candidate, Donald Trump had campaigned on getting rid of the EPA, bringing back “beautiful, clean coal,” overturning the Clean Power Plan, and pulling the US out of the Paris Climate Accord.

The US withdrawal from the Paris Climate Agreement with POTUS’s famous announcement of “so we’re getting out” happened under the assumption that the accord was “very unfair at the highest level” to the US and would undermine the national economy and put the country at a “permanent disadvantage.” However, this was no major shift from the American tradition. At the Earth Summit in 1992, US president George Bush Senior famously declared that “the American way of life is not negotiable.” Five years later, the unanimous US Senate vote (95–0) in favor of the Byrd-Hagel resolution notified the Clinton Administration that no international treaty (including the Kyoto Protocol) imposing GHG emissions reduction on the US while not imposing the same reductions on the developing country parties, or resulting in “serious harm to the economy … including significant job loss, trade disadvantages, increased energy and consumer costs” would be ratified by the Senate. Although, it was George W. Bush who officially discarded any emissions reduction plans for US power plants or any interest in abiding by the Kyoto Protocol. The Democratic Party held the White House, a filibuster-proof majority in the Senate, and a healthy majority in the House of Representatives during 2009–2011, yet their achievements with regards to environmental policy were limited.

On the contrary, the extent to which the conservative populist administration and the Republican controlled legislatures were able to shatter alternative environmental policy arrangements has turned out to be quite broad. Even the Clean Power Plan that seemed more durable was effectively replaced by the Affordable Clean Energy (ACE) rule with much looser emission calculation guidelines. Even though the Clean Power Plan was the outcome of a legal requirement imposed by the Supreme Court on the federal government and therefore beyond the overturning powers of Congress or the president (see MacNeil 2017), the Trump administration was not discouraged from pursuing the enervating federal regulatory procedure for rescinding the EPA’s carbon emission rules. In 2017, Scott Pruitt, the EPA Administrator at the time who had previously sued the agency 13 times as Oklahoma’s attorney general and taken aim at downsizing the “bloated” agency, signed off on the ACE rule, emphasizing the institution’s unfaltering dedication to “writing the wrongs” of the previous administrations by “cleaning the regulatory slate.”

In 2018, US federal budget named “America First: A Budget Blueprint to Make America Great Again” proposed spending cuts for all non-defense/security/veterans affairs departments including the Department of Agriculture (21%), Department of Energy (6%), Department of Housing and Urban Development (13%), Department of the Interior (12%), State Department (28%), and the EPA (31%). With regard to DOE, the proposal would have Fossil Energy R&D, Electricity Delivery and Energy Reliability, and Energy Efficiency and Renewable Energy programs cut fundings to Advanced Research Projects Agency-Energy and the Office of Science; and eliminated Departmental Loan Program (US Department of Energy 2017) that had proven to be very successful in creating manufacturing jobs and establishing renewables (see Homans 2012). Even though the Department of Homeland Security’s budget increased, FEMA faced cuts to some of its grant programs. Although the president ended up reluctantly signing spending bills that were not level with his proposal for State Department allotments, the Congress maintained the prohibition of funding to the Green Climate Fund or supports to initiatives advocating for barring the Export–Import bank and the Overseas Private Investment Corp. from investing in coal technology. Also, no money was appropriated by the Congress to either of the UN’s negotiation and science bodies on climate change, UNFCCC and the IPCC. It is worth noting that many of the decisions in institutions such as the World Bank or USAID are made at levels further downstream than congressional allowance procedures and it somehow takes more than changing the high-level appropriations to entirely choke the flow of money to climate change adaptation and mitigation programs. Yet, these strokes were significant, and the new administration reduced US foreign assistance allocations even further in the years that followed.

In addition to the unwavering rejection of the anthropogenic climate change science, more than 90 environmental regulations with regard to mining and drilling, air and water pollution, biodiversity and land protection, and toxic substances emissions were rolled back or at the very least targeted by the Trump administration, up until August 2020. In a few cases such as the EPA’s attempt to delay a new regulation on methane leaks from oil and gas operations, or the agency’s refusal to announce the designation of areas that meet the National Ambient Air Quality Standards for ozone some states were able to push back in the courts. In a handful of cases, the lawsuits from environmental groups and states successfully reinstated the rolled back rules, for instance, rules concerning mining in Bristol Bay, the use of HFCs, and ozone pollution standards. But overall, the administration successfully transformed the EPA and reversed many important energy and environmental regulations at national and international levels.

### The case of Brazil

Brazil has had one of the most advanced sets of protective environmental legislation and policies in the world (Drummond 1999) despite the post-2018 deregulatory trends.^3^ The historical effectiveness of the Brazil’s environmental policy system can be attributed to a set of different factors. Environmental activism at the national level, the democratization process (Drummond 1999; Jacobs 2002), and the centuries-long struggles of traditional peoples (e.g., indigenous populations and quilombola communities) for survival and against the destruction of natural ecosystems and their ways of living (Carvalho 2000; Toohey 2012; Thorkildsen 2014; Fearnside 2020) have all played a role. Furthermore, the strict environmental laws have been ascribed through a Southern lens to the international pressure from more developed countries aimed at preserving the country as a provider of natural resources and raw materials, hinged upon concepts of environmental colonialism and ecological imperialism (Byrne et al. 2002; Omamo and von Grebmer 2005; Love 2003; Crosby 2015). Despite difficulties in parsing out the most influential factors, it can reasonably be argued that the history of environmental legislation and policy in Brazil has been non-linear and shaped by national interest debates, international forces, and the efforts of the civil society and traditional peoples.

There are major distinction from the US case in the way that Brazil has shifted back and forth between democratic and authoritarian regimes, with unique impacts on its environmental policy institutions.^4^ The transformations of environmental protection structures including laws, decrees, and norms can also be studied from a developmental perspective throughout most of the twentieth century. Up to late1980s, the environment was predominantly perceived as the means for rapid development and national industrialization, without much concern for sustainability. The governmental ideology of that period framed its relationship with the environment as one of extraction of cheap resources required to achieve economic growth at any cost. Under the administration of Getúlio Vargas (1930–1945), especially throughout its dictatorial period (1937–1945), this developmentalist ideology ruled and extended its influence into the short democratic period that followed, reaching the heights of its ascendance during the military dictatorship (1964–1985). The period of redemocratization—roughly between 1981 and 1988—witnessed the consolidation of influential environmental legislation and the institutionalization of fragmented social movements known today as the environmentalist movement. Starting in the late 1980s, bodies of legislation were formed to respond to the rising pollution levels and environmental destruction concerns (Drummond 1999, p. 128).

Despite the unique elements of the Brazilian case stemming from the country’s tumultuous history of political regime shifts, the increasingly partisan qualities of its environmental debate and the resulting congressional deadlock are very similar to the American case. In the same way, the environmental protection system is particular to the political arrangement of the country. Since redemocratization in 1985, the political setup in Brazil has been marked as *coalition presidentialism*.^5^ Compared to the US case, the Brazilian party system is extremely fragmented, with 33 political parties currently registered in the Superior Electoral Court. This context requires the elected executive branch to negotiate agendas with a large array of political forces and form broad alliances so that administrations can aspire to governability and congressional support for project approval and political stability (Avritzer 2019; Abranches 1988). The fragmented model and the brokered distribution of first tier political positions (e.g., ministries) are deemed to have brought about chaos and perpetual crises to the system (Kingstone and Power 2000; Ames 2002), or alternatively, considered an elevating factor for governance stability (Limongi and Figueiredo 1998).

Stability, arguably, held sway during the administrations of Presidents Fernando Henrique Cardoso (1995–2002), Luis Inácio “Lula” da Silva (2003–2010), and the Dilma Roussef’s first term (2011–2014). President Roussef’s second term, however, was short-lived (2015–2016). She lost congressional support and her tenure was interrupted by an impeachment process leading to her removal from office and her vice president Michel Temer taking over through 2018. Her impeachment has occasionally been framed as a *coup d’état* that enabled further entrenchment of neoliberal policies (De Sousa Junior 2017; Tatagiba 2018) just as the 2018 election of the conservative populist Jair Messias Bolsonaro did (Monedero et al. 2019), manifested in reform projects exacerbating social inequality and retrenching welfare systems. The most notable of all have been Temer’s 2017 labor reform and Bolsonaro’s 2019 social security reform concerning workers’ rights and pensions, respectively.

Any analysis of the impact of the recent rise of conservative populism on environmental policy in Brazil needs to be mindful of the decades-long encroaching neoliberalism in the country. Similar to the US, the insertion of neoliberal policy into Brazilian economic model began in the late 1980s. It was not until “the second half of the 1990s,” however, that “the government was determined to put in place a consistent macroeconomic policy framework supporting a new cycle of growth under neoliberalism (Saad-Filho and Morais 2018, p. 70).” Even more progressive administrations—namely former president Lula’s (2003–2010) and Dilma Roussef’s (2011–2016)—under the Workers’ Party (*Partido dos Trabalhadores,* PT) ended up adjusting their initially ambitious goals in accordance with the rising neoliberal wave, limiting the scope of their economic and environmental agenda. In fact, “the looseness of the PT alliance, and Lula’s concessions to neoliberalism, imposed strict limits to his administration, implying that his government would maintain the institutional architecture of mature neoliberalism and follow Cardoso’s economic policies (Saad-Filho and Morais 2018, p. 85).” As an example, the construction of Belo Monte Dam in the Xingu region of the Brazilian Amazon leading to serious negative environmental consequences and displacement of many indigenous population groups (Marin and Da Costa Oliveira 2016) was advanced by Lula’s and accomplished during Roussef’s administration. As another example, it is widely believed that the institutionalization of rural social movements from 2003 to 2016 hindered effective and sustainable land reform in Brazil (Nogueira 2018) while natural land destruction for agribusiness and cropland expansion has always been a main driver of deforestation in the country (Morton et al. 2006).

As demonstrated in the privatization and deregulation animus of the new conservative administration, the neoliberal logic has been elevated from a coercing force to the constitutive driver of policy and government praxis. In the environmental sphere, this animus has acquired an aggressive tone that goes beyond deregulation, occasionally hinting at destruction. In 2018, before taking office, Bolsonaro expressed desire to merge the two ministries of Agriculture and Environment. In practice, this would have most likely resulted in the dismantling of the latter by promoting the position of agribusiness lobbies in determining environmental policy. After strong backlash from the civil society the plan was cancelled. Instead, Ricardo Salles, who was officially being probed for violating multiple environmental laws in favor of businesses was named Minister of the Environment. In agreement with language from the 1992 Rio Declaration, the president affirmed that his administration intended to “protect the environment without, however, creating obstacles to progress.”^6^ Furthermore, he threatened to withdraw Brazil from the Paris Climate Agreement on multiple occasions. While this is yet to take place—partially thanks to the increasing pressure from foreign investors—he is not the only member of his cabinet questioning the reality of climate change and the effectiveness of multilateralism in combatting it. Ernesto Araújo, the foreign minister, endorsing the same anti-scientific discourse has argued that climate change is a form of conspiracy created by “cultural Marxists,”^7^ while the Minister of Agriculture—Tereza Cristina—has labeled climate change “an orchestration from abroad against Brazil” fueled by commercial interests.^8^

The current administration’s plan to address rapid deforestation as one of Brazil's most pressing environmental issues did not go beyond “regularizing the land titles of as many as 97,000 small properties,” summarized in the words of vice president Hamilton Mourão that “land ownership confusion was a major cause of deforestation.”^9^ Environmentalist groups and rural social movements have argued that the suggested land title regularization was rather a reward to invaders and deforesters of public and protected lands. In fact, in the first trimester of 2020, deforestation in the Amazon reached record highs. In April alone, 529 square-kilometers of forest were destroyed, mounting to 171% increase compared to the same month in the previous year.^10^ Available data point to an overall alarming 34% rise in deforestation rates in the Amazon rainforest from August 2019 to July 2020. Simultaneously, the new administration has been systematically undermining environmental supervisory bodies in the country. More than six hundred staffers of the Brazilian Institute of the Environment and Renewable Natural Resources (IBAMA) in an open letter to vice president Mourão, Congress leaders, and the President of the Brazilian Federal Supreme Court (STF) expressed concerns that ignoring the ongoing environmental misconducts would lead to more deforestation, fires, and other forms of environmental destruction.^11^ Even the public focus on the COVID-19 pandemic was regarded by the administration as an “opportunity” and a “moment of relief” that could be exploited to alter the environmental protection system. Although such reforms would mostly pass through the Ministry of the Environment (MMA), in an April 2020 cabinet meeting, the minister of the environment suggested that this “moment of tranquility” deserved a collective effort for *passar a boiada*—“pushing the cattle”—to advance deregulatory infra-legal reforms. Indeed, the MMA successfully made changes to a considerable number of environmental regulations during the months that COVID-19 peaked in Brazil. A report produced by Folha (a large Brazilian media outlet) and Instituto Talanoa identifies a disproportionate increase in deregulation mandates between the months of March and May of 2020. The executive branch published 195 environmental mandates (e.g., normative instructions, decrees, and ordinances ), 12 times the number of environmental mandates the same administration published during the same period in 2019. The use of infra-legal normative instructions and presidential dispatches aimed at undermining the Brazilian environmental protection system can be seen as an attempt by the executive branch to bypass the legislative and change the interpretation of environmental law without having to go through the longer and more costly process of altering the law itself. This is a radical departure from traditional practice over the previous four decades, aligning with the Shock Doctrine argument in which crises are capitalized on as “exciting market opportunities” for abrupt deregulation (Klein 2007, p. 6).

In many cases, civil groups, indigenous populations, and environmental advocacy entities have been able to drag public attention to these changes and challenge some of them in the courts. One example is the MMA’s attempt to grant amnesty to loggers who had committed illegal deforestation in reservations and protected areas that was defeated through legal challenges. Although, an alternative path via the STF was later sought. Another measure suspended by the judiciary was the decree that would have transferred the power of delimiting forest lands from the MMA to the Ministry of Agriculture, stripping the MMA of one of its most important competencies and broadening possibilities for logging activities in permanently protected areas. Many of these attempts faced resistance from the civil society, opposition political parties, and the judiciary. Yet, the executive branch triumphed in approving portions of these normative instructions. For instance, the administration managed to advance normative instruction #4/2020, signed by the MMA that prescribes indemnity in cases of property expropriation within protected land. By altering  the “Declaration of Limits Recognition”, this normative action widens the possibilities for invasion, exploitation, and commercialization of indigenous lands, paving the way for mass evictions of indigenous and quilombola populations—further disenfranchising these communities in favor of mining, logging, and agribusiness. Another mandate—normative instruction #13/2020, also from the MMA—has reduced the minimum legally acceptable distance between populated areas and areas where pesticides can be pulverized, increasing contamination risks for quilombola, indigenous, and rural communities. Infra-legal mandates as such, are not the only avenue of action aimed at effectively dismantling the existing Brazilian environmental protective system. In some cases, high-ranked IBAMA staff were dismissed from employment for not enforcing a more “flexible” environmental approach. As another example, governmental protective activities countering illegal mining on indigenous lands in North Brazil were suspended after negotiations between MMA and major mining companies.^12^

In February 2020, the president submitted a bill to Congress for regulating mining and energy generation activities—mostly through the construction of hydroelectric plants—on indigenous lands. The bill also aimed to legalize tourism and agricultural activities on the same lands.^13^ In fact, beyond the executive efforts, the rise of conservative populism has accelerated the passage of anti-environmental laws through Congress. Sets of proposals condensed into bills (e.g., PL #6299/2002, PL #3729/2004, and PL #6268/2016) designed to loosen or eliminate environmental standard requirements for infrastructure projects, sales of pesticides, wildlife protection; and the constitutional amendment #215/2000 aiming to hamper demarcation measures of new protected lands, all hint at systemic efforts by the conservative movement to dismantle the Brazilian environmental law (Abessa et al. 2019). These efforts could have dire consequences for human health, economy, wildlife, indigenous communities, and global-scale climate change. Brazil, the same country that hosted the Earth Summit in Rio de Janeiro in 1992, voluntarily withdrew from the list of candidates for hosting the 2019 UN Conference on Climate Change (COP25). Similar to the US case, the undermining of environmental regulations in Brazil is almost entirely justified on the pretext of knocking down barriers to economic growth.

## Susceptibility of environmental policy to conservatism

The cases of US and Brazil after 2016 and 2018 presidential elections effectively demonstrate the high vulnerability of environmental policy and sustainability agenda to conservative populist movements. Social and legal structures of environmental protection have largely proven to be *non-resilient* and *unprepared* to weather political storms from the right. That can be partially due to the fact that despite the broad and plastic definition of resilience to the point of “meaninglessness (Klein et al. 2003; Rose 2007),” manmade disaster and shock categories remain narrowly defined within the resilience literature, leaving politics and power relations out of the resilience story. This power-blindness of resilience and ubiquity of apolitical interpretations of the concept, that are not unrelated to the dominance of neoliberal outlooks in the field (Davoudi et al. 2012), obscure the assessment of originators of instability and the way instability travels across systems.

Like any other hegemonic project, neoliberalism rests on both material and ideological foundations. The material basis of neoliberalism does not stand out among other capitalist projects. Capital accumulation and profit maximization guide the political decisions of the actors within the system. Neoliberalism is distinctive, however, at the ideological level. Although there are no clear-cut definitions for neoliberalism, its ideological support lies upon the belief that allocation is more efficient when done through self-regulating markets rather than rent-seeking states that prevent the accumulation of wealth (Friedman 1962; Krueger 1974). For this reason, the long decades of neoliberalism’s rise to a hegemonic position have involved profound reengineering of the state, limiting its field of action and facilitating the market allocation of goods and resources (Brenner and Theodore 2002).

It is in the context of neoliberal hegemony that conservative populist state projects have emerged. Within the neoliberalized global backdrop, communities, towns, cities, and nations must prepare for and adapt to the consequences of the collective decisions of humankind. Local societies with limited resources and power are disproportionately burdened, at least in terms of their relative contribution vis-à-vis global networks of capital, in responding to global social and ecological disasters. Competition for survival constantly sets networks at odds with one another, leading to conflict and instability across other dimensions of socio-spatial relations: territory, place, and scale (Jessop et al. 2008). By channeling development momentum towards more densely populated and wealthier settlements (under the premise that eventually everyone will feel the tide) (Weichhart 2008) and, as a result, escalating socio-spatial gaps at sub-national and national levels, the neoliberal psychology deepens these divides, creating a prolific ground for divisive populist narratives. Although regularly reactionary and often despotic, conservative populist movements need to build political alliances in order to govern effectively (Jessop 2016, pp.71–74). Paradoxically, the nascent political projects, in some cases lacking widespread popular support, need to find allies within the neoliberal hegemonic bloc. Emphasizing environmental deregulation has been a commonly applied strategic tool as an area of agreement in the formation of those alliances.

The cases of Brazil and the US, also illustrate the relevance of material features in the formation of governing alliances. Despite ideological similarities between the hegemonic projects in both countries, local accumulation operates within different institutional environments. For instance, the minimum degree of environmental quality required for accumulation in Brazil has led transnational capital to reject some of Bolsonaro’s environmental deregulation attempts. Moreover, the significant role of social movements in Brazil has further complicated the dismantling of environmental regulation. Those conditions were not necessarily met in the US, leading to the deregulation process facing less resistance, arguably. The ability of conservative populists to build effective alliances and the vulnerability of environmental regulation under neoliberalism is mediated by the specific material conditions for accumulation.

Unlike many other state policies (e.g., trade wars), environmental deregulation would fall in the category of what Gramsci terms *organic* ideology. As opposed to *arbitrary* ideologies, organic ideologies are construed in accordance with the hegemonic vision of the political, economic, and intellectual holders of neoliberalism (Jessop 2016, p.106). For instance, despite the environmental policy subject having become an increasingly partisan point of divide, there is not much fundamental disagreement on the need for environmental policy to be economically profitable or on the superiority of market-based instruments. The fact that environmental deregulation and favorability of market tools are organic to the neoliberal model minimizes the resistance that emerging hegemonic visions with anti-environmentalist agendas face from the existing hegemonic bloc and helps them effectively form new hegemonic alliances. This would leave environmental policy particularly vulnerable to threats lying within the neoliberal realm and an easy prey for emerging conservative populist political forces.

Furthermore, environmental governance has a multilevel structure and is impacted by politics of scales and networks, as state and non-state agents simultaneously act at different scales (Bulkeley 2005). Scalar divisions of power and the resulting conflicts over dominating agendas and subverting competitors also impact the hierarchical relations of scale among networks. Therefore, political surprises, due to their socio-spatial embeddedness, can destabilize networks at different levels by impacting specific nodes or the intra-network positionality of critical nodes. Due to inter-network overlaps, generators of instability can travel between networks and across other dimensions of social space. As a result, durability of environmental policy and resilience of environmental protection structures need to be considered and planned for at local, national, and international levels.

Finally, neoliberalism constantly transforms, emerging in different shades with divergent and overlapping features (Birch and Mykhnenko 2009; McGuirk 2005) that could de-emphasize the need for completely revolutionizing practice. The threat of extreme ideological versions of neoliberalism was overlooked for a long time. That is why the victories of populist conservative movements in the US and Brazil were felt by many political analysists as a *shock*: a form of shock that unlike natural disasters, terrorism, and economic crisis events, there is still no clear set of guidelines to prepare against in the resilience literature. In 2009, Keil wrote that “roll-out neoliberalism as political rhetoric has run its course in many jurisdictions. It is close to impossible for any political party in the current period to win an election with an openly revanchist and neoliberal program. In previous periods of neoliberalization, the roll-out of more market-oriented, less welfarist policy was often greeted with electoral success at various scales: politicians like Rudy Giuliani in New York City, Mike Harris in Ontario or Margaret Thatcher in the UK were voted in on programs that explicitly celebrated ‘revolutionary’ neoliberal shifts. Yet, persuading voters to buy revanchism and privatization under their true label has become a more difficult affair of late. They must be articulated with other political registers to work (Keil 2009, p. 234).” But, considering the post-2016 political developments in the US, UK, and Brazil, it is only fair to ask: Are the roll-out phase of neoliberalization and the “revanchism under its true label” really discredited and rebuffed as economic and political ideologies?

## Conclusion

Politics and power relations determine the influence of market, state, and individual actors in guiding environmental policy design and implementation. The conflicts between these actors constantly breed instabilities. These instabilities could be contained by systems’ structures, or alternatively, force restructuring through disintegration or transformation. Political agency, once dissenting from existing structures instead of reinforcing them, can create instabilities or accelerate disintegration and transformation caused by instabilities created elsewhere. In this article, we discussed what elements of the global neoliberalization trajectory may have led to depletion of natural ecosystems, creation of spaces of neglect and conflict, and the travelling of instability generators through spatial structures, and as a result, opening a prolific gap for populist discourses possibly with anti-environmentalist agendas, without effective resilience mechanisms in place. We also argue that the consensus on profitability as a requirement for environmental conservation and favorability of market tools is one of the major reasons why environmental regulation is vulnerable to onslaughts from the right.

Conservative movements have shown enormous capacity to reshape national and international environmental policy and bring about calamitous blows to the hardly achieved progress over the preceding decades. It is important to evaluate how resilient these achievements and environmental regulations have been against constant pressure from conservative movements. Furthermore, Trump or Bolsonaro electoral victories were not just simple or ordinary changes of administration in favor of conservatives. Even though they rose to power through the democratic process, many scholars and political analysts have marked Trumpism and Bolsonarismo as serious threats to liberal democracy. In both political figures they have identified a willingness to reject democratic rules; toleration or encouragement of violence; denial of rivals’ legitimacy; and a desire to curtail civil liberties (Weizenmann 2019; Hunter and Power 2019). Bolsonaro, for instance, has mentioned in more than one occasion that he would “perform a coup,” “go straight to dictatorship,” and “shut down Brazil’s congress” if necessary (Weizenmann 2019, p. 13). The politics that strongmen such as Trump and Bolsonaro symbolize is not unique to their exclusive dominions. Rodrigo Duterte in the Philippines, Viktor Orbán in Hungary, Narendra Modi in India, and Jarosław Kaczyński in Poland have all posed dangerous challenges to the viability of democracy (Cooper 2021). Yet, by getting trapped within the realms of neoliberalism, many close observers of the political scene got surprised by the emergence of such leaders on the world stage. They failed to acknowledge the risk of extremist movements bolstered and emboldened as a result of the asymmetric development culture, growing inequalities, decaying natural ecosystems, and other generators of instability across the socio-spatial structures.

The lack of preparedness against surprising political dynamics is also partially related to the apolitical themes dominating the resilience literature with a narrow list of manmade shocks and disasters to prepare against. Comprehensive resilience theory would require detailed theorization of political transformation mechanisms and the role of social agency in shaping the trajectory of change in human societies. Building resilience for environmental protection structures increasingly requires having a systemic approach towards the global economy and rigorous mapping of relations of conflict across the globe. The world is a long way from effectively responding to the environmental crisis, and that cannot be done without being mindful of philosophical developments and political ideologies that undermine sustainability. A major initial step towards increasing the endurance of environmental policy in the face of political developments will be to recognize the risks faced by environmental protection structures from populist conservative campaigns.

Finally, political destabilizing shocks are not the only threat to which environmental protection laws or institutions have proven to be vulnerable. Passing environmental policy is hard. But even after promulgation, those policies are subject to failure due to poor design or lack of enforcement. For instance, among US environmental and health and safety regulation, EPA’s water pollution regulations are known to be remarkably successful (Magat and Viscusi 1990), contrary to the agency’s noise pollution abatement efforts (Shapiro 1992). As another example, the EPA fell short in enforcing some critical aspects of the CAA such as its sulfur pollution components. Overall, evaluating the effectiveness of environmental regulation has not received enough attention in the academic literature. Therefore, and also due to lack of coordination and plain-sailing implementation schemes, the environmental sector has failed to communicate a national or international environmental vision to politicians. Critical assessment and following the examples set by successfully implemented environmental laws, as well as laws with higher resilience against political disruptions can streamline and guide the path to effective, enduring environmental regulation. In the political arena, public conversation on environmental policy has stayed mainly limited to the climate change subject and has not been able to escape extremely partisan atmospheres. The solutions of the center and the multilateral international agreements have not met the expectations in curbing climate change and other environmental problems. The environmental and climate change policy of the conservative populist governments struggle with the same catch-22 of their foreign policy. They very much want their countries to shape the international discussion, while withdrawing support from institutions such as the Green Climate Fund leaves them with meaningfully less clout in setting the priorities, especially as they keep denying any major human-caused environmental issues and not offering solutions beyond market instruments. Within this setting, increasing the resilience and endurance of environmental policy requires better understanding of the theoretical bases of shocks and disruptions’ evolution, including political shocks and disruptions, in the context of socio-spatial relations. In addition, it is important to problematize political and social structures of globalized capitalism as opposed to taking its growth logic for granted. Dynamics that breed disruptions may very well reside within the system. The cases discussed in this paper show that addressing the environmental crisis requires building resilience against conservative populist campaigns and, possibly, thinking beyond the traditions of neoliberalism.

## Footnotes

1 It is important to note that the use of the term *populist conservative movements* in this text does not imply a conflation between *populism* and *conservatism*. The focus is on their interaction to explain a specific social phenomenon. Populist movements at their core claim to protect and speak for the people against the elite (Brubaker 2017). Some common elements of populism as an ideologically diverse phenomenon are its discursive logic and amplified communicational, stylistic, and behavioral aesthetics (Laclau 1977, 2005; Taguieff 1995; Kazin 1995; Canovan 2002). This article analyzes the deleterious impacts of a certain branch of populism—the conservative one—for the protective environmental policies promoted by democratic institutions. Populism comprehends heterogeneous political orientations with different possible modes of action, social bases (urban or agrarian), and economics (protectionist, statist, welfarist, or neoliberal) (Brubaker 2017, pp. 357–358).

2 For example, the Trump administration targeted or successfully rolled back at least 90 environmental regulations with regards to mining and drilling, air and water pollution, biodiversity and land protection, and toxic substances emissions.

3 While practical enforcement of protective environmental legislation in a continental-sized country like Brazil has always been challenging, the current administration’s open efforts to undermine successful enforcement have also been impactful. For instance, in 2019, “satellites that detect heat signatures in Brazil issued more than 109,000 fire alerts in the single week from August 13–20, representing a nearly two-fold increase in fires over the prior year,” and despite the decreasing deforestation rates during the previous decade, there was “an especially sharp uptick in deforestation rates in recent years, with sharp increases in both 2019 and 2020 (Abdenur et al. 2020, p. 3).” A report presented at the 2014 UN Climate Change Conference in Germany had previously stated that Brazil’s reduced deforestation and greenhouse gas emissions rates between 2004 and 2009 were unprecedented. Also, in a comparison between 2013 and 1996–2005 deforestation trends, a 70% drop in deforestation and deforestation-associated emissions was identified. In fact, up until mid-2010s, Brazil was internationally regarded as a success story in the fight against illegal deforestation (Correa 2014), corresponding to the Workers’ Party (Partido dos Trabalhadores, PT) administrations under Lula and Dilma Roussef.

4 Drummond argues that the “health” of the Brazilian environmental policy and legislation—which has in part acquired constitutional status—relies on the permanent activism around the subject. Furthermore, he states that the impact and the very possibility of such activism depend upon the democratic permeability of regulatory and executive organs of the republic concerning the environmental issue (Drummond 1999, p. 145).

5 See Abranches, Sergio. 1988. “O presidencialismo de coalizão”, *Dados. Revista de ciências sociais*, número 31.

6 See Ramil, T. (2018, November 11). Bolsonaro diz que Ministérios da Agricultura e do Meio Ambiente devem permancer separados. *Reuters.*

7 See Watts, J. (2018, November 15). Brazil’s new foreign minister believes climate change is a Marxist plot. *The Guardian*.

8 See Phillips, D. (2020, July 27). Resistance to the ‘environment sect’ is a cornerstone of Bolsonaro’s rule. *The Guardian.*

9 See Phillips, D. (2020, July 27); Borges, A. (2020, July 15). Governo vai dar escritura a milhares de ocupações na Amazônia após vistoria a distância. *O Estado de São Paulo.*; and Carvalho, I. (2020, May 22). Salles pediu que governo seja “infralegal” e “passe a boiada”no meio ambiente. *Brasil de Fato.*

10 See Lichterbeck, P. (2020, May 21). O maior crime de Bolsonaro. *Deutsche Welle*.

11 See IBAMA. (2020, July 22). Manifestação Técnica no 2/2020-NMI-CE/DITEC-CE/SUPES-CE.

12 See G1. (2020, August 6). Governo suspende ações contra garimpos ilegais em terra indígena no Pará após visita de ministro. *G1.*

13 See Mazui, G. & L. F. Barbiéri. (2020, February 5). Bolsonaro assina projecto com regras para mineração e gera ção de energia em terras indígenas. *G1*.
